# Increasing the provision of mental health care for vulnerable, disaster-affected people in Bangladesh

**DOI:** 10.1186/1471-2458-14-708

**Published:** 2014-07-10

**Authors:** Nazmun Nahar, Yulia Blomstedt, Beidi Wu, Istiti Kandarina, Laksono Trisnantoro, John Kinsman

**Affiliations:** 1 Protecting Human Rights (PHR) Program, Plan International Bangladesh, Dhaka 1212, Bangladesh; 2 Department of Public Health and Clinical Medicine, Umeå Centre for Global Health Research, Umeå University, 91087 Umeå, Sweden; 3 Universitas Gadjah Mada, Yogyakarta 55281, Indonesia

**Keywords:** Bangladesh, Cyclone, Flood, Disaster, Mental health, Vulnerability, Gender, Poverty, Social determinants of health

## Abstract

**Background:**

Bangladesh has the highest natural disaster mortality rate in the world, with over half a million people lost to disaster events since 1970. Most of these people have died during floods or cyclones, both of which are likely to become more frequent due to global climate change. To date, the government’s post-disaster response strategy has focused, increasingly effectively, on the physical needs of survivors, through the provision of shelter, food and medical care. However, the serious and widespread mental health consequences of natural disasters in Bangladesh have not yet received the attention that they deserve. This Debate article proposes a practical model that will facilitate the provision of comprehensive and effective post-disaster mental health services for vulnerable Bangladeshis on a sustainable basis.

**Discussion:**

A series of socially determined factors render the women and the poor of Bangladesh particularly vulnerable to dying in natural disasters; and, for those who survive, to suffering from some sort of disaster-related mental health illness. For women, this is largely due to the enforced gender separation, or *purdah*, that they endure; while for the poor, it is the fact that they are, by definition, only able to afford to live in the most climatically dangerous, and under-served parts of the country. Although the disasters themselves are brought by nature, therefore, social determinants increase the vulnerability of particular groups to mental illness as a result of them. While deeply entrenched, these determinants are at least partially amenable to change through policy and action.

**Summary:**

In response to the 2004 Indian Ocean tsunami, the World Health Organisation developed a framework for providing mental health and psychosocial support after major disasters, which, we argue, could be adapted to Bangladeshi post-cyclone and post-flood contexts. The framework is community-based, it includes both medical and non-clinical components, and it could be adapted so that women and the poor are actively sought out and provided for. After training, these services could be run by Bangladesh’s pre-existing 50,000-strong Cyclone Preparedness Programme workforce, alongside the country’s extensive network of community-based health workers.

## Background

Bangladesh has the highest natural disaster mortality rate in the world, with over half a million men, women and children lost to disaster events between 1970 and 2005 [1]. The country’s geographical location and low-lying terrain make it particularly vulnerable to two forms of major natural disaster.

The first of these is flooding. Bangladesh is home to the world’s largest delta, formed by the Ganges, Brahmaputra and Meghna rivers. Collectively these rivers drain a total of about 1.7 million square kilometers, including the Himalaya mountains, and they cause flooding in Bangladesh every year. Flooding effectively fertilizes much of the country’s agricultural land, and society has adapted itself to this annual event. However, in extreme years, nearly 70% of the country can be inundated, with serious adverse consequences for millions of people [2].

The second and more dramatic natural disaster that afflicts Bangladesh is the cyclone. Coming in from the south, via the Bay of Bengal, 15 significant cyclones have struck Bangladesh since 1960 [3]. Cyclones striking Bangladesh constitute only around 5% of the global total, but they are the deadliest, accounting for between 80% and 90% of all global cyclone-related losses in terms of lives and property [4]. Over 35 million people live in the coastal zone of the country, all of whom are, to a greater or lesser degree, regularly exposed to these cyclones and their associated storm surges [2].

These geographical factors combine to make the country physically vulnerable to natural disasters, but there are also important social determinants that increase the human impact of major storms and floods [5]. Prime among these is the fact that poverty rates in coastal districts of Bangladesh are significantly above the national average. The majority of coastal district residents are low-income agricultural workers, many of whom are landless and relatively asset-poor [6,7]. Thus, when they are struck by disaster, they have insufficient resources to protect themselves, to adequately rebuild their lives after the event, or to access the medical services that they urgently require. In spite of people’s resilience and adaptive capacity when confronted by adversity [8], this chronic lack of resources can leave the poor facing further vulnerability to future, major weather events.

Unfortunately, the situation is not likely to improve over the coming decades. More than 30 million Bangladeshis are liable to “*lose everything*” because of climate change over the next 30 to 50 years [9]. There is a significant likelihood over this period of increasingly frequent and severe cyclones, as well as heavier and more erratic rainfall, storm surges, sea level rise, and droughts.

The scale of the challenge that lies ahead is therefore immense. Naturally, the primary focus should be on providing for the physical needs of the tens of millions of people who are likely to face these various natural disasters. One issue that receives relatively little attention, however, is the mental health of the survivors. Many survivors endure extraordinary suffering and loss, and a proportion of these require some sort of psychological support to help them to rebuild and continue with their lives.

This Debate article has arisen out of the work of the multi-country ‘INTREC’ programme that examines the social determinants of health in low- and middle-income countries [10]. Our aim here is to highlight the fact that the degree of risk for post-disaster mental illness in Bangladesh is largely determined by social factors, with women and the poor among the most vulnerable. These two groups are currently also systematically under-served by the country’s mental health services. We therefore propose a practical model to provide appropriate and effective post-disaster services for these vulnerable populations on a sustainable basis.

## Discussion

This section builds a case for the institutionalisation of a community-based, post-disaster mental health support system for Bangladesh. We first provide the broad context by describing various significant events in Bangladesh’s political history, after which we present a brief review of some of the major weather-related disasters to have struck Bangladesh over the last 45 years, followed by the post-disaster mental health burdens observed in the country and elsewhere in South Asia after particular events. The policies and actors who are currently engaged in responding to natural disasters are then introduced. Finally, we propose an adaptation of the World Health Organisation’s ‘Framework for Mental Health and Psychosocial Support after Tsunami’ [11] for use after future natural disasters in Bangladesh.

### A brief political history of Bangladesh

After the partition of British India in 1947, Bangladesh became a province of the new state of Pakistan, and was known as East Pakistan. The country’s capital city and main powerbase was in what was then West Pakistan, which corresponds to the current Islamic Republic of Pakistan. There was an unequal division of power between the two ‘wings’ of the country, which did not sit well in the East, and especially so when Urdu was designated as the sole official state language, hitherto spoken only in the West, but now also imposed upon the East.

Anger over this and other political and economic issues led to the emergence in the early 1950s of an independence movement, which finally boiled over in 1970–71 into the Bangladesh Liberation War. As explained further below, a major trigger for this Liberation War was a massive cyclone, the official response to which was seen by many in the East as being completely inadequate. Independence from the West was eventually gained, and the new state of Bangladesh was born in 1971, guided by a constitution which asserted the principles of “*nationalism, socialism, democracy, and secularism*” [12].

Much of the history of Bangladesh has, unfortunately, been characterised by political instability (including a number of coups d’état, two of which resulted in the assassination of the serving head of state), popular unrest, and economic uncertainty. The country has been described as having “*a history of attractive policies and poor implementation*”, which is primarily the result of an absence of political consensus, an inefficient bureaucracy, corruption, and poor management [13]. The material and arguments presented below should be seen within this context.

### Weather-related natural disasters in Bangladesh

The scale of the major weather events that Bangladesh has faced – and in one way or another will continue to face – is briefly illustrated by the following examples of cyclones and floods that have hit the country over the past five decades.

The Bhola cyclone was perhaps the deadliest tropical storm of all time. It struck Bangladesh in November 1970 with a massive storm surge that killed an estimated 300,000 people in coastal areas of the country, and adversely affected the lives of five million more people. Around 100,000 fishing boats, 280,000 cattle, and 400,000 houses were also destroyed in this Category 3 storm [14], which not only left behind immeasurable challenges for the survivors, but, as explained further below, also contributed to major political changes in the country.

A larger, Category 5 storm known as Cyclone Gorky struck Bangladesh in April 1991. This disaster caused around 139,000 deaths in coastal areas and on small offshore islands, with a similar number of people injured. Over 13 million people lived in the affected areas, in some of which over 90% of all structures were destroyed and all livestock were lost. The offshore island of Kutubdia supported a pre-cyclone population of 110,000 people: more than 20,000 died in the disaster. A UN task force put the total cost of reconstruction and rehabilitation after Cyclone Gorky at $1.78 billion [15].

More recently, in November 2007, Cyclone Sidr hit the coastal area of the country just as it was recovering from a huge flood three months earlier. Over 40% of the country’s land area had been flooded, and 1,100 people had died [16]. As Cyclone Sidr approached, 40,000 Red Crescent volunteers were deployed to direct residents from the 15 affected districts to special cyclone and flood shelters. Around 2 million people complied, but there were still around 3,400 confirmed deaths. Ten million people were affected and 1.5 million houses were damaged [1,7]. See Table 1 for details of these and other major cyclones to have struck Bangladesh in the last 50 years.

**Table 1:** Major cyclones that have struck the Bangladesh coast since 1965 [Source: 2; p8]

**Year (Storm name, where given)**	**Maximum wind speed (km/hr)**	**Maximum storm surge height (metres)**	**Death toll**
1965	161	7.6	19,300
1965	217	3.6	870
1966	139	6.7	850
1970 (Bhola)	224	10.0	300,000
1985	154	4.6	11,000
1991 (Gorky)	225	7.6	139,000
1997	232	4.6	150
2007 (Sidr)	223	–	3,360
2009	92	–	190

These disasters did not impact the populations in the affected areas equally. Analysis of the mortality figures from one of the affected areas in the 1991 cyclone, for example, revealed that the death rate was 71 per 1000 among women aged 20–44, as compared to 15 per 1000 for men in the same age group [1]. These figures reflect the gender survival differentials seen in many natural disasters [17]. Some of the reasons for this are likely to be based on the different physiological capacities of women and men to run, swim, or hold on to steady objects, which means that women may be more likely to be swept away by water or high winds [18]. In addition, Bangladeshi women face a socially constructed, gender-specific vulnerability which, it has been convincingly argued, is responsible for a large proportion of this excess mortality [17]. This vulnerability is manifested in a number of ways, as below:

• From childhood, girls and boys learn a number of gender-based behavioral restrictions, and these ensure an enforced separation of women and men throughout their lives. These restrictions are exemplified by *purdah*, which requires, among other things, that women remain within the *bari*, or homestead, unless they are accompanied by a male relative. This can both impede a woman’s access to information about cyclone-induced floods, thereby denying her the opportunity to prepare in advance, while also hindering her ability to re-locate to a safer area before a storm;

• Women shoulder the primary responsibility to look after and protect children – who may cling to their bodies and obstruct their movements – as well as the elderly. This may hamper their efforts to remain safe, and it could also reduce their chances of being rescued;

• The dress codes to which Bangladeshi women must conform, such as the sari, can impede running and swimming [17].

Geography and socio-economic status also interact to determine the extent of an individual’s susceptibility to these storms. As indicated above, poverty rates are higher in these low-lying, climatically vulnerable coastal regions than they are elsewhere in the country, which means that there are simply more poor people in the areas that are likely to be struck by cyclones than there would be if poverty rates were more evenly distributed around the country. By definition, poor people also have relatively fewer resources to prepare for or recover from disasters. Thus, both women and the poor in Bangladesh are at disproportionately greater physical risk from cyclones than other population groups.

Although all of the storms described here were very large, the disaster planning protocols that have been enacted over the past few decades (see below for details) have substantially decreased death rates over time. This means that there are now more survivors than before, which in turn highlights the critical importance of ensuring good mental health support for vulnerable survivors who have experienced trauma and loss.

### Post-cyclone burden of mental health challenges in Bangladesh

The immediate concern after a natural disaster is naturally to ensure that survivors receive the basic necessities to sustain life, such as shelter, food, safe water, and sanitation. But after this acute, emergency phase, many people within the affected populations are left with some level of psychological or mental health problems. These can include post-traumatic stress disorder, depressive symptoms or major depressive disorder, anxiety or generalized anxiety disorder, as well as more general mental health problems such as sleep disruption, substance abuse, and aggression [19,20].

This issue was illustrated by a 1996 study conducted in the Tangail district of Bangladesh, four months after a tornado had killed 600 people. The study assessed the psychological effect of the tornado on people living in the area, as well as their need for psychological assistance, and it found that 66% of the disaster-affected people were traumatized and in need of psychological help [21]. In another survey, conducted two months after Cyclone Sidr in 2007, 25% of 750 survivors were found to have post-traumatic stress disorder, 18% had major depressive disorder, 16% had somatoform disorder, and 15% had a mixed anxiety and depressive disorder [22].

Many studies report a gradual decline over time in the prevalence of disaster-related psychiatric morbidities [23]. However, a population-based survey conducted in south India 4.5 years after the catastrophic Indian Ocean tsunami of 2004 found that 78% of the sample group still had some sort of psychiatric morbidity [24]. While the relative figures for different psychiatric conditions may vary between different disasters, it is nonetheless clear that a significant proportion of the population still require mental health support years after the event.

The overall prevalence of post-disaster mental health illness can be very high, therefore, but disaggregated data show that not all population groups are similarly affected. Two primary risk factors for adverse psychological outcomes after natural disasters have been identified as female gender and low socio-economic status [23]. As shown below, the core drivers of these risk factors are directly or indirectly socially determined and as such, are amenable to change through policy and action. Priority should therefore be given to ensure that women and the poor in Bangladesh are specifically targeted for mental health and psychosocial support after natural disasters.

(a) Female gender

Although there has been an improvement in attitudes towards Bangladeshi women in recent years, and they are now more able to take advantage of economic and social opportunities than they were previously [1], they are still more vulnerable than men through every stage of a disaster [25]. The section above discussed some of the reasons for women’s vulnerability to mortality during extreme weather events. This section examines the reasons why female survivors are more susceptible than men to psychological suffering. The following factors are among those identified as exacerbating women’s difficulty in coping with climate disasters [1], and that may therefore contribute to or facilitate the onset of mental illness:

i) *Limited access to critical services and facilities during and after the disaster*.

a. During the event: even though storm shelters may provide private spaces for women, pregnant women and nursing mothers are often reluctant to share space with strangers, or nurse in front of them.

b. After the event: health services are especially important if a woman herself or her family members are in urgent need of medical care. Even when available, women may not always access health care because of cultural restrictions or household responsibilities [26].

ii) *Household responsibilities*. Once the event is over, there may be unattainable expectations for women to fulfill their family roles and responsibilities [27]. This can set up a vicious circle, as the woman feels guilty because she is struggling with her otherwise regular daily activities, for example due to the loss of utensils and other household essentials. However, she continues to face family expectations, which in turn may cause depression and more guilt [21].

iii) *Sexual harassment*. Women may suffer mental strain when, for example, they are obliged to use public latrines, or if they are seen by men in wet clothing [28]. Sexual harassment of women has also been reported in relief queues while waiting to receive food and other essentials [1].

iv) *The consequences of widowhood*. Men are more likely to suffer bereavement during a natural disaster (because more women die), but the consequences of being widowed are far more serious for a Bangladeshi woman than they are for a man. As it is very difficult for widowed Bangladeshi women to remarry, and over 95% of female-headed households in the country live below the poverty line [29], being widowed in a natural disaster is almost equivalent to being consigned to a lifetime of poverty. As explained below, this is itself a significant risk factor for mental illness.

(b) Socio-economic status

A review of 14 independent studies conducted after different disasters in different countries found that socio-economic status was consistently associated with levels of post-disaster distress, with clear indications that adverse reactions increase as socio-economic status decreases [30]. In Bangladesh, the rural poor who live in low-lying, flood-prone or coastal areas, are most vulnerable to natural disasters. The evidence from this review suggests that they are also inherently at greater risk of acquiring post-disaster mental illness. Furthermore, women make up a disproportionate share of the poor in Bangladesh [31], which is another reason for their increasing vulnerability to mental illness issues for reasons that are entirely socially determined.

### Responding to natural disasters in Bangladesh – programmes and policies

The government’s response to the Bhola disaster of 1970 was heavily criticized, with accusations of gross neglect and charges that the authorities had not acted quickly enough in the aftermath of the storm [32]. The intense frustration felt throughout the country contributed directly to the fall of the government in December 1970. This was followed by a civil war between East and West Pakistan, which triggered an external military intervention by India, and subsequently the birth of Bangladesh as an independent nation in 1971.

Determined not to repeat the same mistake, one of the first major actions of the newly independent Bangladeshi government, in 1972, was to establish the Cyclone Preparedness Programme (CPP). Today this is run by the Ministry of Disaster Management & Relief in collaboration with the Bangladesh Red Crescent Society. The CPP’s vision is to “*minimize loss of lives and properties in cyclonic disaster by strengthening the capacity in disaster management of the coastal people of Bangladesh*”. This work is accomplished by around 50,000 trained volunteers who, among other things, build cyclone shelters, assist with evacuation and rescue, conduct first aid and emergency relief work, and also support survivors after cyclones [33].

At the time of Cyclone Sidr in 2007, nearly 4,000 cyclone shelters – multi-storied buildings, raised above ground-level on concrete pillars so as to resist storm surges – were operational. A study conducted two months after the storm reported no fatalities among people who managed to move into a public cyclone shelter [7]. After the storm, the volunteer workforce was deployed to distribute food and safe drinking water. Meanwhile, health worker teams implemented appropriate health care intervention measures [20,34]. The affected populations appreciated the disaster effort, though it is important to note that many also said they would have liked volunteers and health workers to stay longer in the cyclone-impacted areas [34].

The relatively low number of deaths caused by Cyclone Sidr is widely considered to have been a result of the government’s timely cyclone forecast and early warnings, the successful evacuation of coastal residents from the storm’s projected path, and appropriate post-storm emergency relief [7].

Thus, a strong framework for reducing mortality and disease from cyclones and floods has been established in Bangladesh. However, in spite of the significant needs identified in the previous section, the post-disaster mental health and psychosocial support for affected populations provided today remains very limited, as outlined below:

i) The *National Plan for Disaster Management, 2010–2015*, is a detailed 100-page document that includes just one action point (out of 133 action points) related to mental health. This action point aims to “*Enhance recovery schemes including psycho-social training programmes in order to mitigate the psychological damage of vulnerable populations, particularly children, the elderly and the disabled, in the aftermath of disasters*” [2], p80. This is of course welcome, but two issues stand out. First, these few lines constitute the entire focus on mental health, which clearly suggests that this is not yet seen as a major disaster-related priority. Second, in spite of the post-disaster mental health challenges facing women and the poor, as discussed above, neither are given any special priority in the National Plan.

ii) The *Bangladesh Climate Change Strategy and Action Plan*[35] provides a detailed roadmap outlining the country’s response to climate change. One of the core pillars of action identified in the document aims to ensure ‘*Food security, social protection and health*’; but no specific mention is made of mental health.

iii) The country’s *Health Policy*[36] constitutes the basis for all health-related activities conducted in the country and provides guidance on, among other areas, primary and emergency health care. There is no mention of mental health in the policy. Although a draft Mental Health Act was produced several years ago, it has yet to be passed through Parliament and enacted. Meanwhile, the Lunacy Act of 1912, which permits discrimination against the mentally ill, still remains on the statute books [37].

iv) Health expenditure in Bangladesh was 3.7% of GDP in 2011 [38], of which just 0.44% is spent on mental health. In addition, there is no social insurance scheme coverage for mental disorders, and there is only one mental health hospital in the whole of the country. Bangladesh’s 161 million people are served by just 90 psychiatrists [39]. Rural residents suffer more from the lack of mental health services than urban residents, even though they live in the very places where disaster-related mental health care needs are the greatest.

To summarize, just as specific population groups face increased risk of mental illness for reasons that are socially determined, the concomitant lack of post-disaster mental health care for these same people is also socially determined. There is a clear and entirely appropriate focus from the government and from the country’s development partners to provide emergency relief after natural disasters, but the serious psychological and mental health consequences of natural disasters have not yet received the attention that they deserve in Bangladesh. The reasons for this have been identified as a lack of awareness and/or a lack of interest from the government [9,21]. In other words, although the disasters themselves are brought by nature, addressing the human cost of them is hampered purely by socially determined factors.

### Proposal for a new approach – applying the WHO’s ‘Framework for Mental Health and Psychosocial Support after Tsunami’

The frequency and intensity of floods and cyclones is expected to rise in Bangladesh as a result of climate change. With that, the burden of mental illness is also expected to increase in the country. There are therefore strong grounds to mainstream mental health issues into disaster response in a more comprehensive fashion [40].

Some key principles have been identified as essential to providing an appropriate and sustainable mental health response after natural disasters. One is that the mental health teams should be aware of the socio-economic status, culture, traditions, language and local livelihood patterns of the populations that they are working with. In other words, the teams must include local, suitably trained people. The evidence presented in this paper also indicates that special care and attention should be given to those whose social conditions make them most vulnerable to mental health problems – women and the poor – both in terms of how to physically make contact with them, and in terms of the specific support that they may require. Further, to facilitate programme sustainability, the teams should be integrated into any local network of governmental and non-governmental organizations already present in the area [21]. This means that services need to be provided through both primary health care and community settings [41].

**Figure 1:**
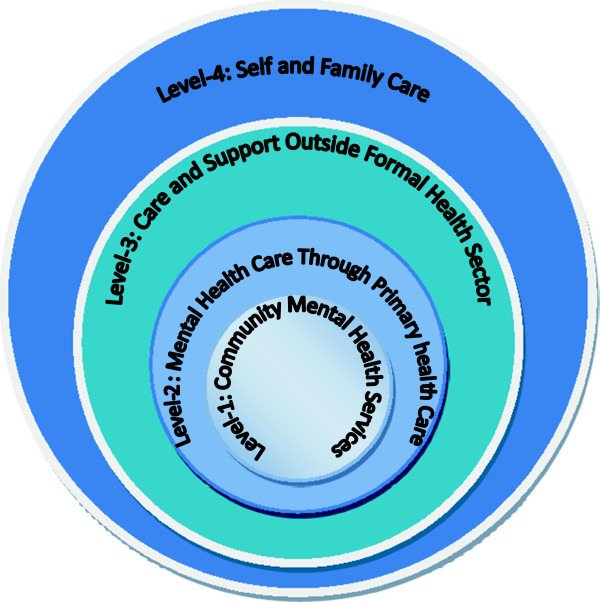
**Ideal mix of mental health services within each District **[11]**, page 14.**

A useful conceptual model along these lines has been created out of the experience of the catastrophic Indian Ocean tsunami of December 2004, in which approximately 230,000 people died. This propelled WHO to build a framework for the provision of mental health and psychosocial support for survivors of major natural disasters in the future [11]. This model could be applied to mental health care and support in post-disaster situations in Bangladesh – again, with a particular focus on women and the poor. The model, which includes four different levels, is presented graphically in Figure 1.

One of the model’s essential premises is that post-disaster mental health interventions need to cover both clinical interventions (including medication and psychotherapy) and basic, non-clinical, psychological support interventions (such as psychological first aid). Clinical interventions are required for only a small proportion of disaster-affected populations. Indeed, although the destruction brought about by the 2004 tsunami caused great distress in the majority of the population, the increase in mental disorders that required psychiatric help was estimated to be about 5-10%. Thus there was no need for medical, psychiatric interventions for the great majority of the population [11]. Rather, this majority would benefit from a range of non-medical, social, and basic psychological interventions that can help to reduce distress.

The work at Levels 1 and 2 (Community Mental Health Services; and Mental Health Care through Primary Health Care) should be led by community mental health teams on a mobile or outreach basis, with hospital support as necessary. The teams should ideally consist of 3–4 staff (including one doctor, accompanied by a constellation of nurses, nurse aides or community health workers), some of whom are locally recruited to ensure awareness and understanding of the prevailing cultural norms. Women should constitute at least half of the team, whose members should also have good skills in non-medical and psychosocial support.

Levels 3 and 4 (Care and Support outside the formal health sector; and Self and Family care) focus on the training of traditional healers, teachers, religious leaders, women leaders and other community leaders to provide support; as well as strengthening community networks through activities that facilitate isolated people to meet one another and generate mutual support. WHO estimates that up to 50% of survivors may have psychosocial needs that could benefit from this sort of community-based approach [11].

Such a combination of mental health care services goes somewhat further than the level of services called for in the recently developed ‘Balanced Care Model’ for mental health care in low-income settings such as Bangladesh [42]. The Balanced Care Model describes the need for services at the primary care level, and for more advanced services wherever specialist staff may be available. There is no specific mention made of community-based services; and, as discussed above, specialist mental health care workers are in any case almost completely absent in rural Bangladesh.

The innovation, as well as the potential key to success for implementation of the WHO’s post-disaster mental health care framework in Bangladesh, therefore, is the Cyclone Preparedness Programme’s nearly 50,000 dedicated volunteers [33], as well as the many others in the country who work within the health sector and who provide services to the community on a door-to-door basis. Collectively, this constitutes a large, pre-existing workforce which could be appropriately trained in non-medical psychosocial support, and who would be available to assist at all four of the model’s Levels immediately after a disaster.

The intervention could perhaps follow an approach adopted in Orissa state in India, after a major cyclone killed around 10,000 people in 1999. After the storm, a team of psychiatrists and psychologists made regular visits to one of the affected areas. Their visits were supported by local volunteers, such as imams, community leaders, and teachers. The volunteers received training in supportive psychotherapy which included empathy, reassurance, listening patiently, and guidance. They also set up short-stay homes for orphans and widows from the storm, as well as self-help groups for mutual support. The intervention was positively evaluated (albeit in a non-randomised, non-controlled form [43]), and could form the basis for similar approaches in Bangladesh, which could then be more rigorously evaluated and further improved.

It would be important to follow current best practice guidelines while developing the post-disaster intervention strategy for Bangladesh. The TENTS guidelines, for example, were produced in 2008 by the European Network for Traumatic Stress, and they were designed to assist in the provision of effective psychosocial care following disasters [44]. TENTS provides generic advice, and is not specific to low-income settings, but the core principles may still provide useful guidance. These include consideration of planning, preparation and management; general components of the response (concerning, for example, human rights and cultural issues); and specific components of the response that may be relevant during different phases after the event, from the first week to the period beyond three months.

There is both sufficient manpower and an existing infrastructure in Bangladesh into which post-disaster mental health services, based on the WHO’s framework for the provision of mental health and psychosocial support for survivors of major natural disasters, could be integrated. On this basis, there is a good foundation for maximizing the possibilities for programmatic sustainability [45], and for delivering an effective post-disaster mental health support service for the most vulnerable people in the country.

## Summary

This Debate paper has argued for a realignment of post-disaster assistance in Bangladesh. In addition to the broadly effective work already conducted by the Cyclone Preparedness Programme, there is also a need to respond to the socially determined vulnerability of particular categories of people – specifically women and the poor – and to place a much stronger emphasis on mental health care and support for these groups. The Cyclone Preparedness Programme has greatly reduced mortality rates in natural disasters, but a large proportion of the survivors – and especially women and the poor – are deeply traumatized and need psychosocial support. Up until now, the provision of such support has been inadequate. With the likely increase in severity and frequency of major weather events in Bangladesh due to climate change, this issue should be addressed as a matter of urgency.

As a step towards this, we have proposed the adoption of a mental health and psychosocial support framework developed by WHO after the 2004 Indian Ocean tsunami. This would be applicable to the post-cyclone and post-flood context of Bangladesh, and it would include both clinical and non-medical, social approaches to supporting traumatized and vulnerable survivors. The key to success would be the ability to train and support the unique, pre-existing potential workforce for the non-medical component – the 50,000-strong workforce of Cyclone Preparedness Programme volunteers – alongside the country’s extensive network of community-based health workers, who could provide simple clinical services as necessary.

In conclusion, we urge the Government of Bangladesh and the country’s development partners to join together in adopting WHO’s ‘Framework for Mental Health and Psychosocial Support after Tsunami’ as part of a comprehensive post-disaster mental healthcare package.

## Abbreviations

CPP: Cyclone preparedness programme; GDP: Gross domestic product; INTREC: INDEPTH training and research centres of excellence; WHO: World Health Organisation.

## Competing interests

The authors declare that they have no competing interests.

## Authors’ contributions

NN had the idea for the study and led the first draft of the manuscript. BW contributed to the first draft of the manuscript. YB, IK and LT participated in the design of the study and helped to draft the manuscript. JK coordinated the process and finalized the manuscript. All authors read and approved the final manuscript.

## Pre-publication history

The pre-publication history for this paper can be accessed here:

http://www.biomedcentral.com/1471-2458/14/708/prepub
